# Upregulation of ADAR Promotes Breast Cancer Progression and Serves as a Potential Therapeutic Target

**DOI:** 10.1155/2021/2012903

**Published:** 2021-09-27

**Authors:** Xiao Li, Guangshun Sun, Liangliang Wu, Guoqiang Sun, Ye Cheng, Jing Tao, Zhouxiao Li, Weiwei Tang, Hanjin Wang

**Affiliations:** ^1^Department of General Surgery, Nanjing First Hospital, Nanjing Medical University, Nanjing, Jiangsu, China; ^2^Department of General Surgery, Nanjing Drum Tower Hospital, The Affiliated Hospital of Nanjing University Medical School, Nanjing, Jiangsu, China; ^3^Department of General Surgery, The Fourth Affiliated Hospital of Nanjing Medical University, Nanjing Medical University, Nanjing, Jiangsu, China; ^4^Department of Hand Surgery, Plastic Surgery and Aesthetic Surgery, Ludwig-Maximilians University, Munich, Germany; ^5^Hepatobiliary/Liver Transplantation Center, The First Affiliated Hospital of Nanjing Medical University, Key Laboratory of Living Donor Transplantation, Chinese Academy of Medical Sciences, Nanjing, Jiangsu, China

## Abstract

**Background:**

Breast cancer (BC) is the most common cause of cancer death worldwide, and its incidence is increasing every year. This study aims to investigate the expression characteristics of ADAR gene in breast cancer and to explore its role in the occurrence and development of BC and its possible mechanism.

**Methods:**

TCGA portal was used to detect the expression of ADAR in cancer including BC, and its correlation with clinicopathological data as well as other genes was analyzed via UALCAN database. The TISCH database evaluated the expression of ADAR in different types of cell populations in BC at the single-cell level. The Kaplan–Meier plotter database was used to predict the correlation between ADAR expression and BC patient prognosis. The Human Protein Atlas was used to detect the expression of ADAR in tissues and location of ADAR mRNA in cells. Moreover, the relationships between immune response and ADAR expression in BC were assessed with the use of the TISIDB. Metascape and STRING were applied to predict ADAR with other protein interactions. Finally, the effect generated by ADAR expression on cell proliferating, invading, and migrating processes was assessed in vitro with knockdown and overexpression strategies.

**Results:**

ADAR was significantly upregulated in BC tissues compared to paracancerous tissues. Single-cell RNA analysis showed that ADAR was specifically upregulated in cancer cell clusters and was also expressed in stromal and immune cell clusters. The upregulation of ADAR was positively correlated with clinicopathological stage and negatively correlated with BC prognosis. Experimental processes in vitro revealed ADAR knockdown hindered, proliferated, invaded, and migrated levels of BC cells, whereas over expression of ADAR played the opposite effect. ADAR protein, which may interact with OASL, STAT2, and IFIT3, was mainly located in the nucleoli in cells and primarily involved DNA modification and apoptotic signaling pathway. Immune factors may interact with ADAR in BC, and ADAR was found noticeably linked with immunosuppressor such as IL10, CD274, and IDO1.

**Conclusion:**

ADAR is significantly upregulated in breast cancer tissues, which may promote the progression of BC through the interaction of cancer cells, stromal cells, and immune cells. Targeting ADAR may offer new hope in treating breast cancer.

## 1. Introduction

Breast cancer (BC) is the most common malignancy among women in the world and the leading cause of death among women aged 40–55 in developed countries, including China [1]. In the past decades, the incidence of human BC has been on the rise and continues to increase, posing a great threat to women's lives. According to statistics collected by the American Cancer Society, there will be more than 271,000 new cases of BC and about 42,260 deaths in 2019 [2]. Being a heterogeneous disease, BC can be classified into several main subclasses based on the expression status of oestrogen receptor (ER), progesterone receptor (PR), human epidermal growth factor receptor (HER2), and antigen ki-67 (Ki-67) [3]. In recent years, many advances have been made in the diagnosis and treatment of BC. However, many patients have low chemotherapy efficiency and eventually become resistant to endocrine therapy after long-term treatment. In many cases of BC, disease progression and death may eventually occur. Therefore, there is an urgent need to discover the mechanisms of BC occurrence and development and to identify biomarkers for its diagnosis and treatment.

Adenosine deaminases acting on RNA (ADARs) are dsRNA-editing enzymes that catalyze the conversion of adenosine (A) to inosine (I). Inosine shows similar properties as guanosine. As a result, A-to-I editing has a great impact on edited RNAs. RNA editing is a basic modification event that bypasses genomic information to control nucleotide changes. In higher eukaryotes, the most characteristic and ubiquitous type of RNA editing is A-to-I editing [4]. ADAR enzymes were originally thought to work only in the coding regions of certain genes. However, high-throughput sequencing has shown that A-to-I editing most often targets noncoding sequences [5]. Recently, many editing sites in the micro-RNA transcriptome have been discovered [6, 7]. The overall biological significance of ADARs is still largely unknown. External stimuli can affect A-to-I editing mediated by ADAR, indicating that ADARs can quickly regulate a wide range of coding and RNA. The imbalance of ADAR expression or activity can lead to a variety of diseases, such as cancer, Aicardi-Goutieres syndrome, and amyotrophic lateral sclerosis [8].

However, predecessors have reported on the role of ADAR in BC, for example, Sagredo et al. reported that ADAR plays an important role in BC progression through the regulation of mRNA stability impacting the viability of BC cells [9]. However, previous studies have limited the study of ADAR in BC, and there is no systematic study of its functions, such as its global expression in BC tissues, its location and function in single cancer cells, and its relationship with the tumor microenvironment. Through a series of bioinformatic predictions and specific cell experiments, this study explored the expression of ADAR in BC, especially in cancer cell populations, its clinical relevance, possible molecular mechanisms, and its relationship with the tumor microenvironment. This research provides a new potential diagnostic and therapeutic target for BC and provides preliminary ideas for further research and pharmaceuticals of ADAR in the future.

## 2. Materials and Methods

### 2.1. ADAR Expression Level Analysis and Clinicopathological Analysis

In order to show the databases involved in the research more clearly, we have compiled Table 1. TCGA portal was used to investigate the expression of ADAR in different tumor tissues and corresponding paracarcinoma tissues. The Human Protein Atlas database was used to examine ADAR expression in different tissues. UALCAN refers to a comprehensive and interactive web resource to delve into cancer OMICS data, which was used here to compare ADAR expression in BC patients of different races, ages, and molecular subtypes. Tumor Immune Single-Cell Hub (TISCH) is a single-cell RNA-sequencing (scRNA-seq) database focusing on tumor microenvironment, which provides detailed cell-type annotation at the single-cell level, enabling the exploration of TME across different cancer types including BC.

### 2.2. ADAR Expression Level and Survival Analysis

Kaplan–Meier plotter was used to compare correlations between ADAR expression and overall survival (OS), first progression (FP), distant metastasis-free survival (DMFS), and postprogression survival (PPS). The Kaplan–Meier survival plot was used to compare the two groups of cases, and hazard rates with log rank *P* values and 95% confidence intervals were acquired.

### 2.3. Tools for ADAR Location in Cells

The Human Protein Atlas, compiling numerous reports and tissue, Cell and Pathology Atlas forms, and gene data in cells and tissues were utilized for obtaining ADAR location in cells.

### 2.4. Interaction and Functional Enrichment Analysis of ADAR

Metascape, a web portal, combines 40 independent knowledgebases's functional enrichment, interactome analysis, gene annotation, and membership search. It promotes comparative analysis of multiple independent and orthogonal experiments across datasets. STRING is a database that can be used to search for protein-protein interactions, including direct and indirect connections. The conclusions obtained are comprehensively calculated and predicted, and knowledge transfer between organisms and interactions are summarized in other (main) databases. We used Metascape and STRING to create an interaction network between ADAR and other important proteins as well as pathways. TCGA portal was also used to investigate the association between ADAR and other genes in BC.

### 2.5. Tool for Immune-Related Analysis of ADAR

TISIDB, a web portal for tumor and immune system interactions, integrating a range of heterogeneous data, was adopted for delving into the Spearman correlations between ADAR and immune-modulator expression.

### 2.6. RNA Isolation, Reverse Transcription, and Quantitative Real-Time Polymerase Chain Reaction

Overall RNA according to tissues under pairing received the extraction with TRIzol reagent (Thermo Fisher Scientific, Waltham, MA, USA), and overall RNA inside plasma received the extraction with the use of TIANamp Virus RNA tool by complying with the guidelines of the producer. Based on the Prime-Script™ RT-PCR tool (Takara, Dalian, China) by complying with the guidelines of the producer, we acquired overall complementing DNAs (cDNAs). ADAR expressing state was determined by qRT-PCR with the primer pair, i.e., ADAR-F 5′-ACCAGGTGAGTTTCGAGCC-3′; ADAR-R 5′-TCTTGTAGGGTGAACACCGTG-3′. Glyceraldehyde 3-phosphate dehydrogenase (GAPDH) became one internal control. Glyceraldehyde 3-phosphate dehydrogenase (GAPDH) was as a control.

### 2.7. Cell Culturing and Transfecting Processes

MCF-7 and ZR75-1 cells received the culturing process using RPMI 1640 medium (BI, USA), involving 10% fetal bovine serum (FBS) (Gibco, USA) under 37°C inside one 5% CO_2_ chamber covering streptomycin (100 mg/mL) and penicillin (100 IU/mL). SiRNA (si-NC) and small interfering RNA against ADAR (si-ADAR) were produced by Hongxin Company (Nanjing, China). We conducted transfecting process based on the use of the Opti-MEM (Gibco, USA) and the solution RNAi Fectin™ (cat. G073, Abcam, Canada). The study acquired cells for exploring the information to carry out 48 h posttransfecting process. ADAR (human) siRNA: 5′-GGAUGCAAAUCAAGAGAAATT-3′.

### 2.8. Plasmids Constructing and Transfecting Process

Hongxin Biological Technology Co., Ltd. (Jiangsu, China) offered the control plasmid vector pcDNA3, as well as the ADAR overexpression plasmid pcDNA3-ADAR. Plasmid pcDNA3- ADAR overexpressed, using pcDNA3.1 (+) to be vector, involving a human ADAR structure-related cDNA gene complete length and received Hind III and EcoR I digesting process. Nearly, 1 × 10^6^ cells received the seeding process inside 6-well plates and the 24 h culturing process. Following the manufacturer's guidelines, cells were transfected using 2 *μ*g plasmid in serum-free medium based on DNA Fectin^TM^ Plus (cat. G2500, AbCAM, Canada). After 12–16 h, serum-free medium was converted as complete medium, and 10% FBS was added. Subsequently, the transfected cells can be used for further experimental procedures.

### 2.9. Cell Proliferation Experiments

In the clone forming experimental process, cells under transfection received the seeding inside 6-well plates at 1000 cells per well density and subsequently the culturing process inside RPMI 1640 medium involving 10% FBS. After 10 days, the cells received the fixing based on the use of methanol, followed by the staining with GIMSA. Eventually, colonies received imaging and counting. Specific to cell counting kit-8 (CCK-8) testing process, MCF-7 and ZR75-1 cells first received the transfecting and incubating processes at 37°C. Next, CCK-8 solution (Biosharp, China) received the introduction inside the respective well and the 2-hour incubating process. The absorbance received the measuring process at 0, 24, and 48 h at 450 nm.

### 2.10. Transwell Migration and Invasion Assays

By complying with the producer's guidelines, MCF-7 and ZR75-1 cells received seeding in the upper chambers with 200 *μ*l of serum-free RPMI 1640 medium. The transwell chamber (Corning, NY, USA) received paving process with Matrigel mix (BD Biosciences, San Jose, CA, USA) for invasion testing process and with no use of Matrigel mix for migration tests. RPMI 1640 medium and 10% FBS received the introduction inside the bottom chamber to be a BC cell chemoattractant. When the 24 h incubating process was achieved, the upper chambers received the fixing, followed by staining based on crystal violet (Kaigen, Nanjing, China) for 15 min. For the visualizing procedure, the cell lines received the photograph and count procedures inside 5 fields.

### 2.11. Wound Healing Assay

MCF-7 and ZR75-1 cells received the transfecting process when the seeding on 6-well culture plates was achieved. With a standard 20 *μ*l pipette tip, artificial linear wounds received elimination on the fused cell monolayer. Free floating cells and debris isolated inside the well bottom received slow removal. The medium received the introduction, and the plate received the incubating process at 37°C. The scratch gap width received the recording based on one inverted microscope, followed by photographing at 0, 24, and 48 h. The respective experimental process was in triplicate for the distinctions of the quantitative cell migrating width as well as the original wound width.

### 2.12. Statistics-Related Analyzing Process

The continuing information received comparative analysis by performing one individual *t*-testing process of the 2 groups. Statistics-related analyzing process was performed with SPSS (version 22.0, IBM, USA) and presented graphically in GraphPad Prism 8.0. A *P* value of 0.05 was considered to be statistically significant.

## 3. Result

### 3.1. Expression of ADAR in BC Tissues

**Figure 1:**
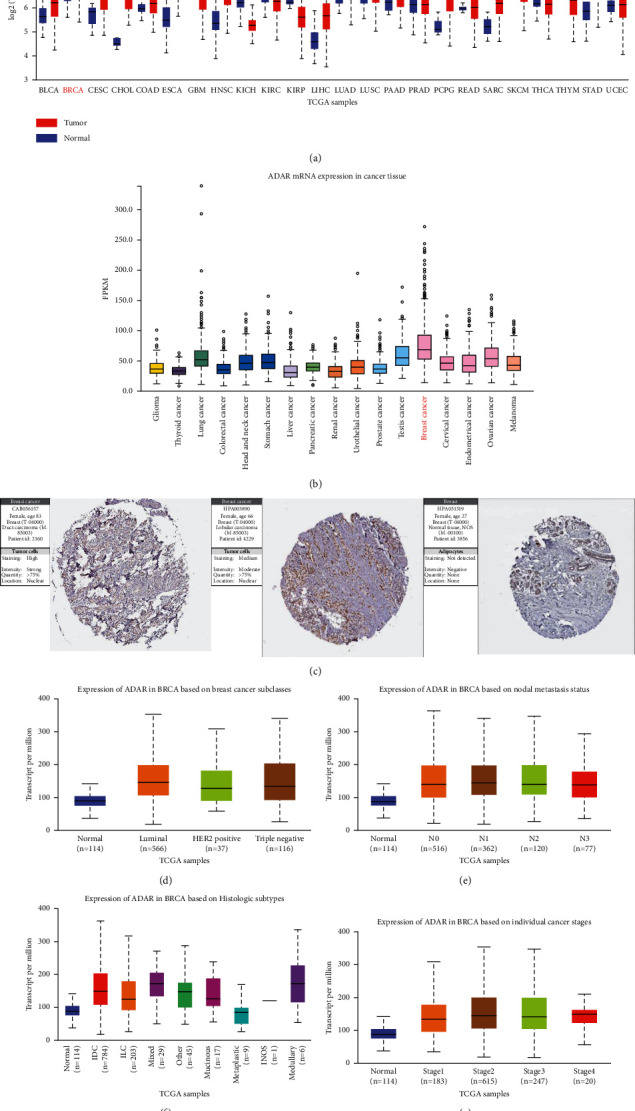
Expression of ADAR in BC tissues. (a) The expression level of ADAR mRNA in different types of cancer tissues compared to normal tissue. (b) The expression level of ADAR mRNA in different types of cancers. (c) The protein expression level of ADAR in BC tissue and normal breast tissues. (d–g) The correlation between ADAR mRNA expression and breast cancer subtypes, tumor stage, lymph node metastatic status, and histological subtypes was analyzed. The Wilcoxon rank sum test was used to assess the significance of observed differences.

The TCGA portal showed that the expression of ADAR in tumor tissues was obviously higher than that in normal tissues (Figure 1(a)). The expression level of ADAR mRNA in different types of cancer tissues indicated that the expression level of ADAR in breast cancer tissues was also higher than that in other cancers (Figure 1(b)). Analysis of Human Protein Atlas data indicated that ADAR staining was stronger in BC tissue than in normal breast tissue (Figure 1(c)). Subgroup analysis based on breast cancer subtypes, lymph node metastasis, histological subtypes, and individual cancer stages showed that ADAR mRNA levels in BC patients were significantly higher than those in normal tissues (Figures 1(d)–1(g)).

### 3.2. Clinical Role of ADAR in BC

**Figure 2:**
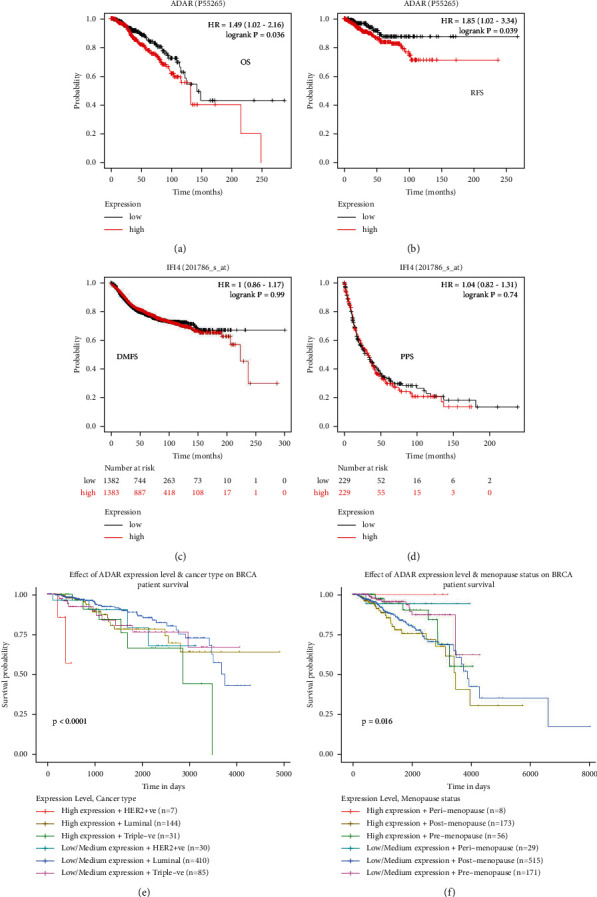
Clinical role of ADAR in BC. (a–d) The relationship between ADAR expression and BC patients (OS, overall survival; RFS, relapse-free survival; DMFS, distant metastasis-free survival; PPS, postprogression survival). (e, f) The relationship between ADAR expression and different BC cancer types and menopause status.

The prognostic potential of ADAR in BC was further examined using Kaplan–Meier plotter. The results showed that patients with higher ADAR expression had lower OS and RFS, but there was no significant correlation with DMFS and PPS (Figures 2(a)–2(d)). TCGA database showed that ADAR expression was associated with different BC cancer type and menopause status (Figures 2(e) and 2(f)).

### 3.3. ADAR Plays a Promoting Role in BC Cells In Vitro

**Figure 3:**
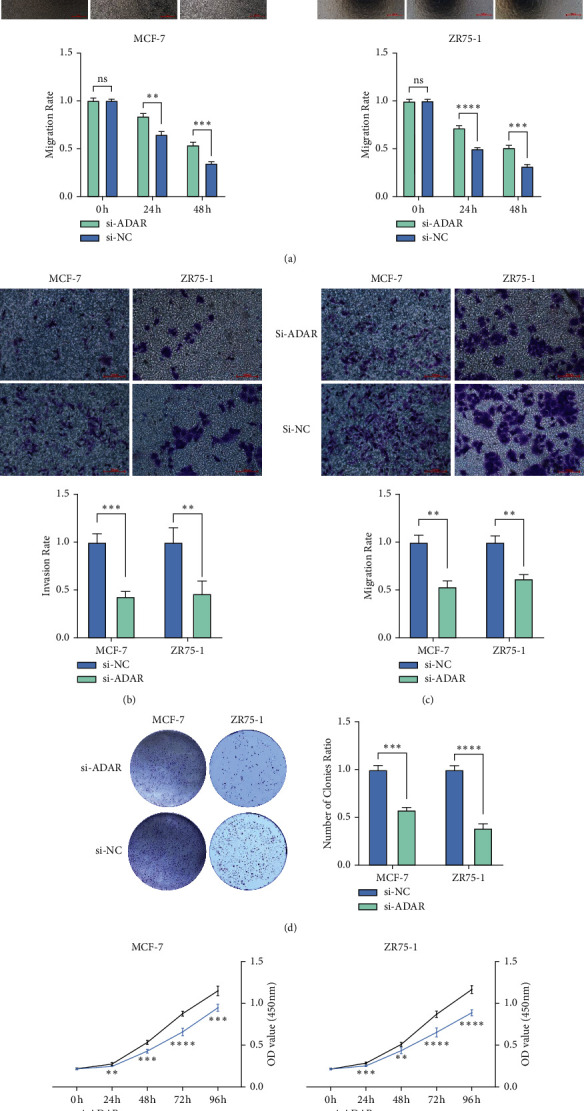
Knockdown of ADAR could inhibit the development of BC cells. (a) Scratch assay of knockdown of ADAR. (b) Knockdown of ADAR could inhibit the migration of BC cells. (c) Knockdown of ADAR could inhibit the invasion of BC cells. (d) Plate cloning experiment of knockdown of ADAR. (e) CCK8 results showed that suppression of ADAR by si-ADAR exhibited a slower proliferation. ^*∗∗*^*P* < 0.01; ^*∗∗∗*^*P* < 0.001;  and ^*∗∗∗∗*^*P* < 0.0001.

**Figure 4:**
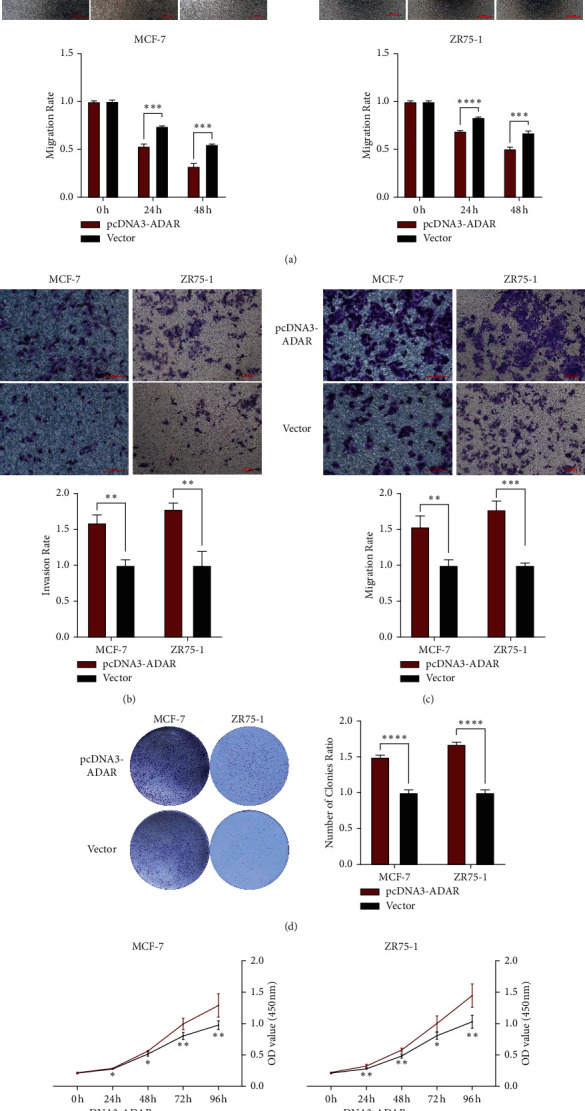
Overexpression of ADAR could promote the development of BC cells. (a) Scratch assay of overexpression of ADAR. (b) Overexpression of ADAR could promote the migration of BC cells. (c) Overexpression of ADAR could promote the invasion of BC cells. (d) Plate cloning experiment of overexpression of ADAR. (e) CCK8 results showed that overexpression of ADAR exhibited a higher proliferation ^*∗*^*P* < 0.05;  ^*∗∗*^*P* < 0.01;  ^*∗∗∗*^*P* < 0.001;  and ^*∗∗∗∗*^*P* < 0.0001.

The results of scratch assay showed that, in breast cancer cell lines, the scratch closure rate of inhibiting ADAR was significantly lower than that of the control group (Figure 3(a)). Compared with the control group in the confluence monolayer transwell experiment of cultured breast cancer cell line, si-ADAR inhibited the relative migration and invasion rate of ADAR (Figures 3(b) and 3(c)). Plate cloning and CCK-8 assay showed that ADAR gene knockdown significantly inhibited the proliferation of MCF-7 and ZR751 cells compared with the control group (Figures 3(d) and 3(e)). Overexpression of ADAR plays the opposite effect (Figures 4(a)–4(e)). These results suggest that inhibition of ADAR can delay the proliferation, invasion, and migration of breast cancer in vitro.

### 3.4. Genes and Proteins Cointeracted with ADAR Are Associated with DNA Modification and Apoptotic Signaling Pathway

**Figure 5:**
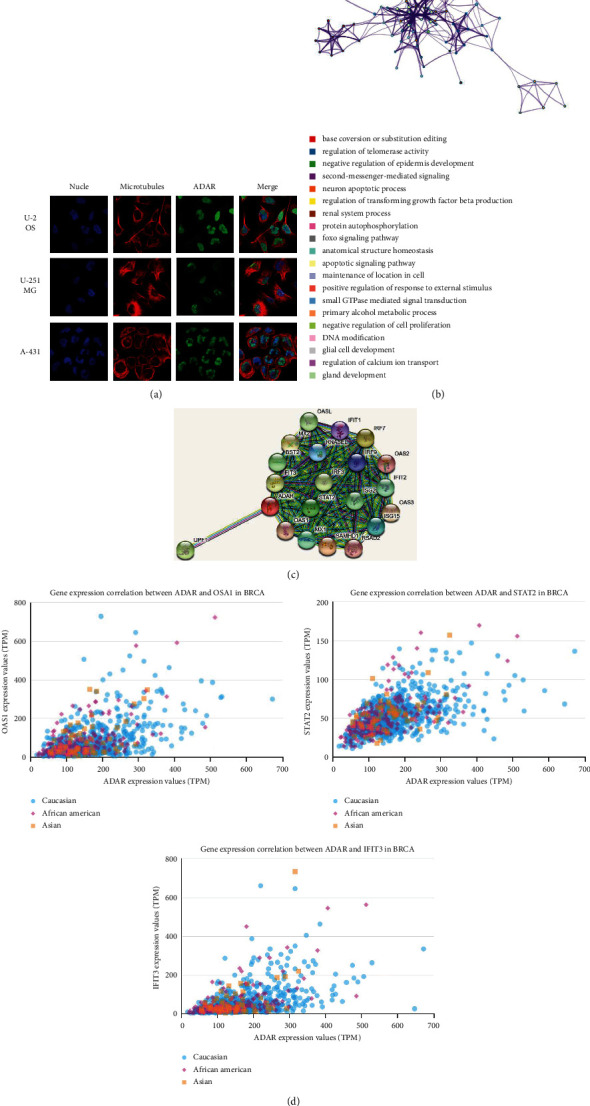
Genes and proteins cointeracted with ADAR are associated with DNA modification and apoptotic signaling pathway. (a) ADAR located in the nucleus. (b) Pathway analysis of ADAR enrichment. The network of enriched terms is colored by cluster ID. Nodes that share the same cluster ID are typically close to each other. (c) Interactions between ADAR and other proteins. (d) Relationship analysis between ADAR and OASL, STAT2 and IFIT3 in different ethnic groups.

According to the Human Protein Atlas database, ADAR is located in the nucleoli in U-2 OS, U-251 MG, and A-431 cells (Figure 5(a)). Enrichment analysis of coexpression genes performed using Metascape indicated that ADAR was primarily involved DNA modification and apoptotic signaling pathway (Figure 5(b)). A STRING interactive network was used to identify proteins which can interact with ADAR (Figure 5(c)). And, further research showed strong relationship between the expression of ADAR and the proteins which might interact with ADAR such as OASL, STAT2, and IFIT3 (Figure 5(d)).

### 3.5. Research Results of ADAR at Single-Cell Level

**Figure 6:**
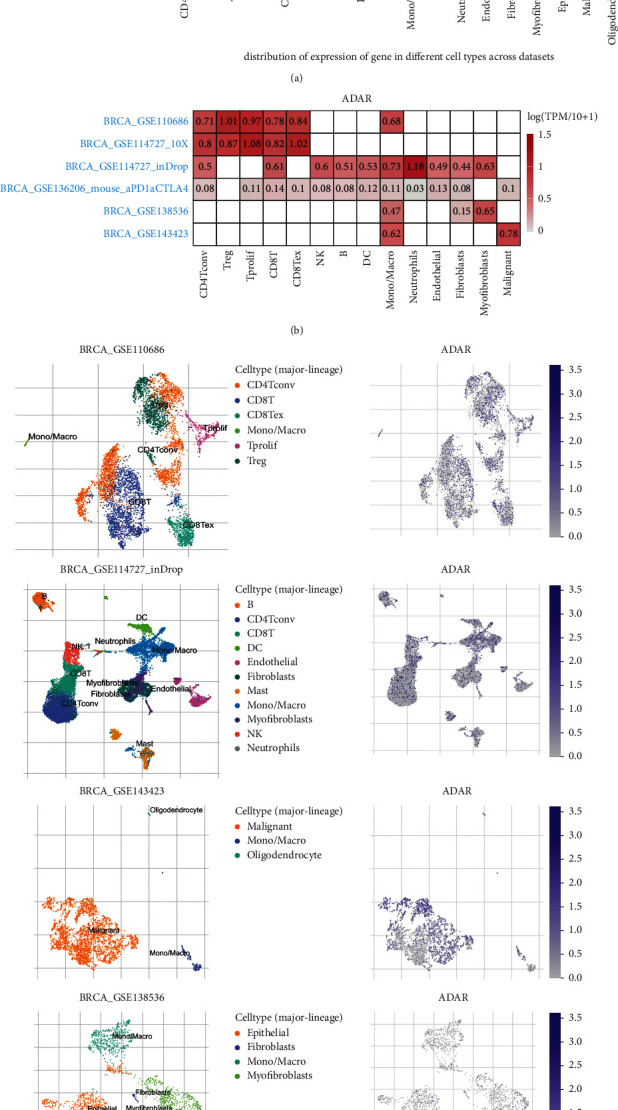
Research results of ADAR at single-cell level. (a) Violin diagram displays the distribution of ADAR expression in different cells from different databases. (b) Heatmap displays the value of ADAR expression in different cells from different databases. (c) Single-cell cluster map of ADAR in different databases.

We studied the expression of ADAR at the single-cell level. Figures 6(a) and 6(b) showed the distribution of ADAR expression in different databases. Further data indicated that the ADAR was mainly expressed at the CD4 Tconv, CD8T, CD8Tex, and Treg cell clusters in database BRCA-GSE110686. In database BRCA-GSE114727, the ADAR was mainly expressed at the CD4 Tconv, CD8T, and DC cell clusters. In database BRCA-GSE143423, the ADAR was enriched at the malignant, mono-, macro-, and oligodendrocyte cell clusters. In the BRCA-GSE138536 database, ADAR was mainly concentrated in epithelial cells, fibroblasts, and mono-/macro-cell clusters (Figure 6(c)). These results suggest that ADAR may also function in stromal cells or immune cells other than cancer cells.

### 3.6. ADAR Expression Was Correlated with Immune Factors

**Figure 7:**
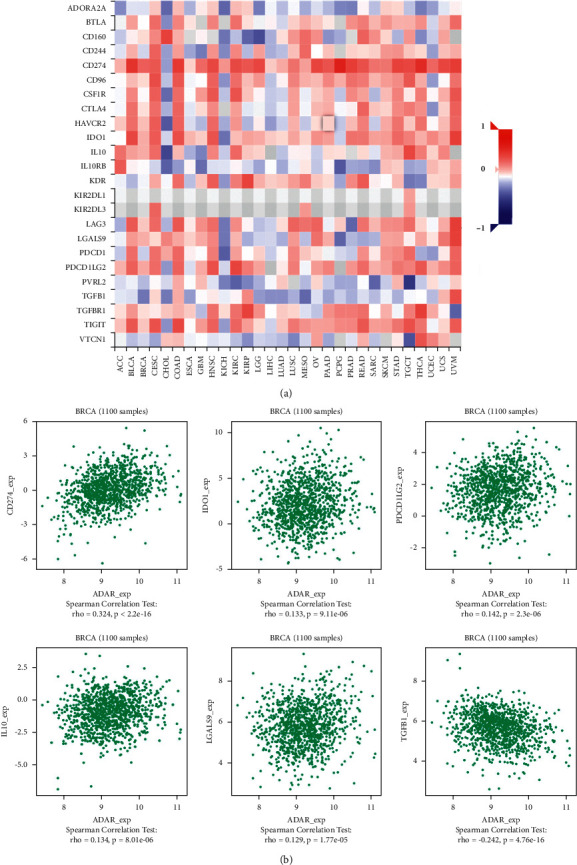
Correlation between ADAR expression and immunoinhibitors in BC. (a) The heat map shows the correlation between ADAR and immunosuppressive factors in different cancers. (b) Line graph shows the correlation of ADAR with specific immune indicators in BC.

**Figure 8:**
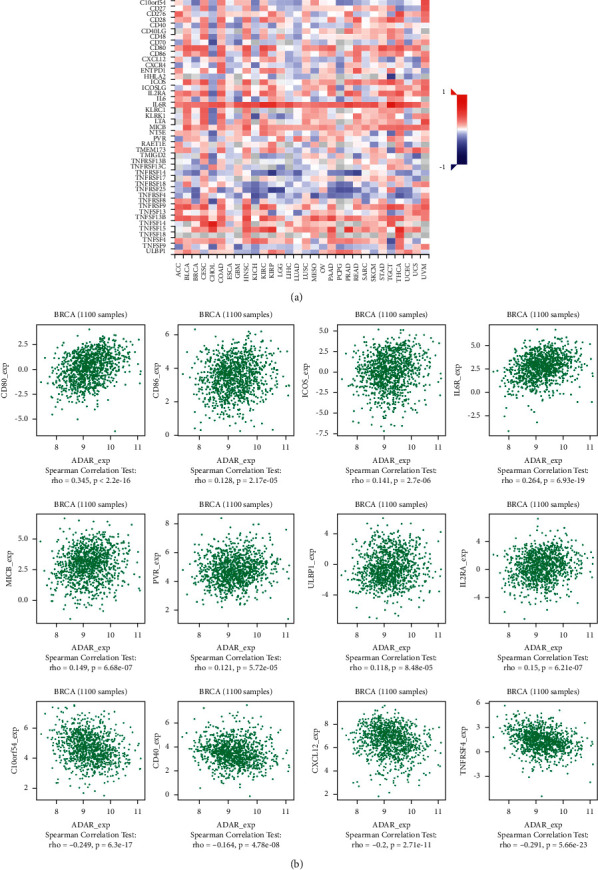
Correlation between ADAR expression and immunostimulators in BC. (a) The heat map shows the correlation between ADAR and immunostimulators factors in different cancers. (b) Line graph shows the correlation of ADAR with specific immune indicators in BC.

**Figure 9:**
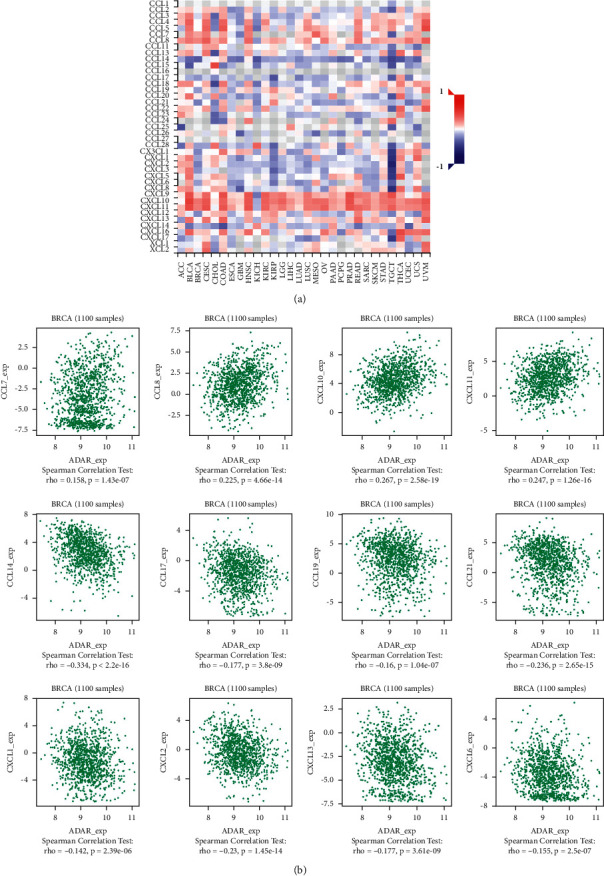
Correlation between ADAR expression and chemokines in BC. (a) The heat map shows the correlation between ADAR and chemokines in different cancers. (b) Line graph shows the correlation of ADAR with specific immune indicators in BC.

Existing studies have confirmed that the immune system is closely related to the occurrence and development of tumors. Therefore, we studied the relationship between the expression of ADAR and immune factors. As shown in Figures 7–9, there was a strong correlation between the expression of immunoinhibitors (CD274, IDO1, IL10, etc.), immunostimulators (CD80, CD86, ICOS, etc.), and chemokines (CCL7, CCL8, CXCL10, etc.) and the expression of ADAR.

## 4. Discussion

Recent research studies have reported the role of ADAR in promoting gastric cancer, pancreatic cancer, thyroid cancer, and other cancers [10, 11]. In this study, we found that ADAR was significantly upregulated in BC tissues compared to paracancerous tissues. Subgroup analysis based on breast cancer subtypes, lymph node metastasis, histological subtypes, and individual cancer stages showed that ADAR mRNA levels in BC patients were significantly higher than those in normal tissues. Patients with higher ADAR expression had worse prognosis. These results are consistent with Sagredo et al.'s study [12].

Single-cell RNA-sequencing (scRNA-seq) presents better insights into cell behavior in the context of a complex tumor microenvironment [13, 14]. Interestingly, in this research, single-cell analysis showed that ADAR was specifically upregulated in cancer cell clusters and was also expressed in stromal cells (such as fibroblasts) and immune cells (such as macrophages). This is the first report of ADAR expression in various cell clusters for cancer research. As for ADAR and stromal cells, Takeda et al. reported that both ADAR1 expression and AZIN1 RNA editing levels were significantly elevated in colorectal cancer (CRC) tissues vs. normal mucosa, and these findings correlated with the increased expression of mesenchymal markers, vimentin and fibroblast activation protein. Intriguingly, ADAR1 expression was specifically upregulated in both cancer cells and fibroblasts from cancerous lesions. Conditioned medium from cancer cells led to induction of ADAR1 expression and activation of AZIN1 RNA editing in fibroblasts [15].

As for ADAR and immune cells, Pujantell et al. demonstrated the role of ADAR1 in regulating innate immune function in primary macrophages, suggesting that macrophages may play an essential role in disease associated with ADAR1 dysfunction. They also showed that viral inhibition was exclusively dependent on innate immune activation consequence of ADAR1 knockdown, pointing towards ADAR1 as a potential target to boost antiviral immune response [16]. Baal et al. established conditional murine CD11c Cre-mediated ADAR1 gene ablation, which did not induce general apoptosis in CD11c cells but instead manifests in cell type-specific effects in DC subpopulations. ADAR1 deficiency resulted in a preferential systemic loss of CD8/CD103 DCs, revealing critical dependency on ADAR1, whereas other DC subpopulations were moderately affected or unaffected. These results revealed an unrecognized role of ADAR1 in DC subset homeostasis and unveiled the cell type-specific effects of RNA editing [17]. In this study, we have unearthed many immunoinhibitors related to ADAR, such as IDO1, PDCD1, and CD274, which are positively correlated with the expression of ADAR. In addition to immunoinhibitors, we also found that some immunostimulators such as CD80, CD86, and ICOS and chemokiness such as CCL7, CCL8, and CXCL10 are all positively correlated with the expression of ADAR. It is suggested that ADAR plays an important role in immune regulation in BC. Therefore, the combination of inhibitors against these immunological checkpoints and ADAR inhibitors may potentially enhance the anticancer effect in patients with BC.

In this study, we simply verified the function of ADAR in cancer cells. Experimental processes in vitro revealed ADAR knockdown hindered, proliferated, invaded, and migrated levels of BC cells, whereas overexpression of ADAR played the opposite effect. ADAR protein, which may interact with OASL, STAT2, and IFIT3, was mainly located in the nucleoli in cells and primarily involved DNA modification and apoptotic signaling pathway. Kung et al. reported that expression of ADAR1, specifically its p150 isoform, was required for the survival of triple-negative breast cancer (TNBC) cell lines. In TNBC cells, knockdown of ADAR1 attenuated proliferation and tumorigenesis. Moreover, ADAR1 knockdown led to robust translational repression. ADAR1-dependent TNBC cell lines also exhibited elevated IFN-stimulated gene expression. IFNAR1 reduction significantly rescued the proliferate defects of ADAR1 loss. These findings established ADAR1 as a novel therapeutic target for TNBC tumors [18]. Ding et al. reported that 8-Cl-Ado inhibited cell proliferation and induced G1 phase arrest and apoptosis at least by targeting the ADAR1/p53/p21 signaling pathway [19]. The findings may provide us with insights into the role of ADAR1 in breast cancer progression and help us better understand the effects of 8-Cl-Ado in the treatment of breast cancer. We also confirmed the correlation between ADAR and OASL, STAT2, and IFIT3 in BC, which implies their positive correlation. Previous studies have shown that high mRNA expression of OASL and OAS3 was associated with poor prognosis in all breast cancer patients [20]. Other studies have revealed that OASL was regulated by upstream long noncoding RNA TINCR to promote the metastasis of breast cancer [21]. Combined with these studies, it is suggested that ADAR may be involved in the OASL-related regulatory pathway.

This paper systematically studies the relationship between ADAR and BC, but there are still many shortcomings. First, only the expression and function of ADAR in BC cancer cells were verified, and the functions of ADAR in other cells such as immune cells and stromal cells were not studied. Second, there is a lack of animal studies to further test efficacy in vivo. Third, no attempt was made to design inhibitors for the domain of ADAR to inhibit the function of ADAR. Finally, there are many types of breast cancer, and this study generally verified the function of ADAR in MCF-7 and ZR75-1 cells, without subdividing the types. We are looking forward to further report on the relationship between ADAR function in different cell types and cancers.

## 5. Conclusion

ADAR is significantly upregulated in breast cancer tissues, which may promote the progression of BC through the interaction of cancer cells, stromal cells, and immune cells. Targeting ADAR may offer new hope in treating breast cancer.

## Figures and Tables

**Table 1:** Summary of databases used in this study.

Name	Link
TCGA portal	http://www.tcgaportal.org
UALCAN	http://ualcan.path.uab.edu/
Human Protein Atlas	https://www.proteinatlas.org/
Kaplan–Meier plotter	http://kmplot.com/analysis/index.php?p=background
Metascape	http://metascape.org/gp/index.html#/main/step1
STRING	https://string-db.org/cgi/input.pl
TISIDB	http://cis.hku.hk/TISIDB/index.php
TISCH	http://tisch.comp-genomics.org/home/

## Data Availability

The data used to support the findings of this study are available from the corresponding author upon request.
